# Dictionary of Practical Medicine

**Published:** 1845-06

**Authors:** 


### DICTIONARY OF PRACTICAL MEDICINE.

This valuable work, we are glad to perceive, has reached its sixth monthly part, and is going on steadily to its completion. No publication of the times has commanded more universal or higher admiration than Copland's Dictionary, and this sentiment of the press, we are persuaded, will be warmly responded to by the profession all over the country. We are obliged to improve, as a profession, with such works as the Cyclopædia, and the Dictionary of Practical Medicine, in general circulation. As works of reference they are invaluable. No physician who has had an opportunity of consulting them will feel that he can get along without one or the other of them, and all who are able will enrich their libraries with both. Y.

